# Energetic Films Realized by Encapsulating Copper Azide in Silicon-Based Carbon Nanotube Arrays with Higher Electrostatic Safety

**DOI:** 10.3390/mi11060575

**Published:** 2020-06-06

**Authors:** Xuwen Liu, Yan Hu, Hai Wei, Bingwen Chen, Yinghua Ye, Ruiqi Shen

**Affiliations:** 1 School of Chemical Engineering, Nanjing university of Science and Technology, Nanjing 210094, China; lxw@njust.edu.cn (X.L.); haiweinjust@gmail.com (H.W.); bingwenchennjust@gmail.com (B.C.); yyinghua@njust.edu.cn (Y.Y.); rqshen@njust.edu.cn (R.S.); 2 Micro-Nano Energetic Devices Key Laboratory, Ministry of Industry and Information Technology, Nanjing 210094, China

**Keywords:** energetic films, composite energetic materials, Cu(N_3_)_2_@carbon nanotubes (CNTs), electrostatic sensitivity, electrochemical deposition

## Abstract

Since copper azide (Cu(N_3_)_2_) has high electrostatic sensitivity and is difficult to be practically applied, silicon-based Cu(N_3_)_2_@carbon nanotubes (CNTs) composite energetic films with higher electrostatic safety were fabricated, which can be compatible with micro-electro mechanical systems (MEMS). First, a silicon-based porous alumina film was prepared by a modified two-step anodic oxidation method. Next, CNTs were grown in pores of the silicon-based porous alumina film by chemical vapor deposition. Then, copper nanoparticles were deposited in CNTs by electrochemical deposition and oxidized to Cu(N_3_)_2_ by gaseous hydrogen azide. The morphology and composition of the prepared silicon-based Cu(N_3_)_2_@CNTs energetic films were characterized by field emission scanning electron microscopy (FESEM), transmission electron microscopy (TEM) and X-ray diffraction (XRD), respectively. The electrostatic sensitivity of the composite energetic film was tested by the Bruceton method. The thermal decomposition kinetics of the composite energetic films were studied by differential scanning calorimetry (DSC). The results show that the exothermic peak of the silicon-based Cu(N_3_)_2_@CNTs composite energetic film is at the temperature of 210.95 °C, its electrostatic sensitivity is significantly less than that of Cu(N_3_)_2_ and its 50% ignition energy is about 4.0 mJ. The energetic film shows good electric explosion characteristics and is successfully ignited by laser.

## 1. Introduction

In recent years, micro-electro mechanical systems (MEMS)-related technologies have been widely used to fabricate miniature safe systems and micro-initiators [1,2,3,4]. The currently used lead-based primary explosives can hardly meet the requirements of the micro-initiator for energy due to the miniaturization of the ignition device. There is an urgent need to find an environmentally friendly substitute with higher safety and energy density [5,6,7,8,9,10]. Energetic films can be integrated directly on the ignitor without manipulating dangerous reactants [11,12], which makes the process safe and attractive for commercial products. Copper azide (Cu(N_3_)_2_) is a promising alternative to lead-containing primary explosives. Compared with lead azide and lead styphnate, the reaction product of copper azide has little harm to the environment. Hence, it is a green and environmentally friendly primary explosive [13,14,15]. In addition, copper azide has excellent initiation performance, and its limit charge of initiating pentaerythritol tetranitrate (PETN) is just 1/6 of that of lead azide [16,17]. In MEMS pyrotechnics, using copper azide as the initial charge can reduce the volume occupied by energetic charges while ensuring detonation performance, and is in line with the trend of miniaturization of modern pyrotechnics. However, the high electrostatic sensitivity of copper azide restricts its practical application [18,19].

Carbon nanotubes (CNTs) as a new type of one-dimensional nanomaterial with excellent electrical conductivity, heat transfer properties and mechanical properties have shown important application values in the field of energetic materials [20,21]. Rubtsov et al. [22] added a small amount of carbon nanotubes to high-energy explosives such as trinitrotoluene, pentaerythritol tetranitrate and benzotrifuran nitrogen oxides (TNT, PETN, BTF) through recrystallization, reducing the electrostatic sensitivity of explosives. Compared with pure octogen (HMX), the impact and friction sensitivities of the carbon nanotubes/HMX nanocomposites synthesized by Li et al. [23] have been reduced by 73% and 29%, respectively. Doping of carbon materials can effectively reduce the electrostatic sensitivity of copper azide. Pelletier et al. [24] filled copper oxide particles into oriented carbon nanotubes with open ends prepared using a self-supporting alumina film as a template to reduce copper oxide to copper in a hydrogen atmosphere. Then, copper was azidated by hydrogen azide gas. In order to simplify the preparation of Cu(N_3_)_2_@CNTs/Al_2_O_3_ composites, Wang [25] and Zhang [26] deposited copper in oriented carbon nanotubes by electrochemical deposition directly. At present, the research of carbon nanotube composite energetic materials mainly focuses on mixing carbon nanotubes with energetic materials mechanically. The composite energetic material obtained in this way cannot give full play to the advantage of the unique structure of carbon nanotubes. The work of filling energetic materials into carbon nanotubes is still rare, and the templates for preparing carbon nanotubes are all self-supporting alumina films. The nano-energetic materials prepared in this way and the micro energetic devices prepared on the basis cannot be compatible with MEMS-related technologies.

In recent years, materials such as silicon and silicon carbide which have excellent properties of corrosion resistance, high-temperature and high-pressure resistance have been widely and maturely applied in MEMS-related technology [27]. In order to apply Cu(N_3_)_2_ to the energetic charge of MEMS pyrotechnic products, a method for preparing silicon-based Cu(N_3_)_2_@CNTs composite energetic films with higher electrostatic safety and compatibility with MEMS technology was developed in this work. The micromorphology, crystal structure, composition and thermochemical properties of the material were studied. In the process of preparing silicon-based Cu(N_3_)_2_@CNTs composite energetic films, it is very important to prepare porous alumina films with a large pore size, straight and orderly channels and no barrier layer on the silicon substrate. It is related to the success of the subsequent deposition of aligned carbon nanotube arrays in the template. In addition, the processes for depositing Cu nanoparticles in aligned carbon nanotubes by electrochemical deposition are also key factors that determine the density of copper azide in carbon nanotubes.

## 2. Materials and Methods 

Materials: phosphoric acid, oxalic acid, copper sulfate pentahydrate, boric acid, sodium hydroxide, sodium azide, nitric acid, absolute ethanol and thiourea. The reagents above are all analytical reagents. Sinopharm Group Chemical Reagent Co., Ltd. (Shanghai, China); pure water: homemade. Nanjing Yipuyida Technology Development Co., Ltd. (Nanjing, China); hydrogen, argon and acetylene. The purity is 99.97%, 99.99% and 99.9%, respectively, Shanghai Pujiang Special Gas Co., Ltd. (Shanghai, China).

Warning of the hazards: Sodium azide is highly toxic and explosive. When it reacts with acid, it will produce hydrogen azide gas, which is also explosive. In this work, copper nanoparticles were reacted with hydrogen azide gas to produce copper azide with high energy density as the primary explosive. These azides are very dangerous, so they must be dealt with carefully during the experiment.

The silicon wafer was placed in acetone, isopropano and ultrapure water sequentially for ultrasonic cleaning and dried with nitrogen. The electron beam evaporation method was used to deposit Ti and Al layers on the silicon wafer. First, we adjusted the current to stabilize the filament current to 4.5 A and the voltage to 9 kV. During this process, the pressure raised slightly, indicating that the electron beam hit the evaporation material and the film began to deposit. The Ti layer served as a transition layer to increase the strength of the adhesion between the Al layer and the silicon substrate.

**Figure 1:**
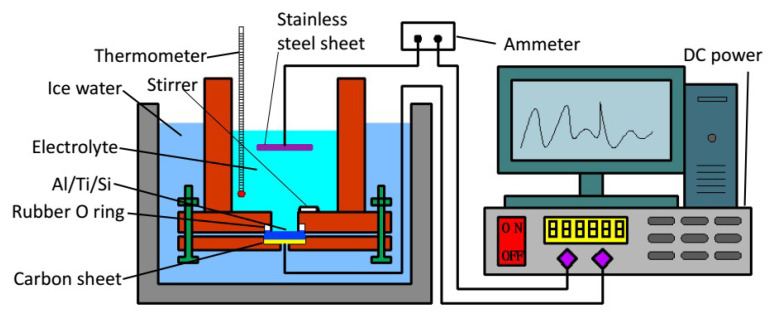
Schematic diagram of an anodizing device.

An improved two-step anodic oxidation method [28] was used to prepare the silicon-based porous alumina film, the device is shown in Figure 1. The preparation process can mainly be divided into three steps:

First, 0.3 mol/L oxalic acid was used as the electrolyte, and ethanol/water (*v*/*v* = 1:1) as the electrolyte solvent; we kept the temperature of the electrolyte at 1–3 °C, and increased the voltage from 0 to 100 V; the speed of magnetic stirring was 430 r·min^−1^, and the time of the first step of anodizing the silicon-based aluminum layer was 40 min;

Next, the sample was immersed in a mixed solution of 1.8% CrO_3_ and 6% phosphoric acid at 65 °C for 3 h to remove the alumina film formed, and then the second step of anodizing was performed, the electrolyte was 0.5% phosphoric acid solution;

Finally, the sample was immersed in a 5% phosphoric acid solution for 40 min at 30 °C. The purpose of this step was to enlarge the diameter of the pores.

An aligned carbon nanotube array was prepared in a silicon-based porous alumina film channel using a chemical vapor deposition method. The silicon-based porous alumina film was placed in a tube furnace, and the temperature was raised to 700 °C under vacuum conditions, while a mixed gas of acetylene, hydrogen and argon was passed in. The vacuum degree was kept at 0.05 MPa, and the deposition time was 90 min. Then, the copper nanoparticles were deposited in the aligned carbon nanotubes by an electrochemical deposition method. The composite film was the working electrode and the platinum electrode was the counter electrode. The electrolytic solution was 0.08 mol/L copper sulfate pentahydrate and 0.4 mol/L boric acid, then thiourea or polyvinylpyrrolidone (PVP) were added as an additive and the current density was 0.1 mA·cm^−2^. The effects of different additives on the deposition of copper nanoparticles in the carbon nanotubes were explored. Finally, the sample was immersed in a 0.5 mol/L sodium hydroxide solution for 1 h to remove the aluminum oxide film. We left it in the air for 24 h, and then the oxidation of the sample was characterized.

At normal temperature, nitric acid reacts with sodium azide to produce hydrogen azide gas. The gas–solid reaction of copper with hydrogen azide can produce copper azide. The reaction equation involved in the above process is as follows:

HNO_3_ + NaN_3_→HN_3_ + NaNO_3_ (1)

Cu + 3HN_3_→Cu(N_3_)_2_ + NH_3_ + N_2_ (2)

The silicon-based Cu_2_O@CNTs composite film and Cu@CNTs composite film were placed in a closed container. We passed N_2_ into it to remove the air and added 4 g·mL^−1^ of nitric acid and 2 g·mL^−1^ of sodium azide solution. After 72 h of reaction, a silicon-based Cu(N_3_)_2_@CNTs composite energetic film was obtained.

Field emission scanning electron microscopy (FESEM) and transmission electron microscopy (TEM) were used to characterize the micro-morphology of the samples and X-ray diffraction (XRD), high-resolution transmission electron microscopy (HRTEM), selective electron diffraction (SAED) and energy dispersive X-ray spectroscopy (EDS) were used to characterize the crystal structure and composition. The thermal decomposition kinetics of the Cu(N_3_)_2_@CNTs composite energetic film were studied by differential scanning calorimetry (DSC).

The performance of the energetic films was tested by laser ignition experiments, and high-speed photography was used to record the ignition process. By changing the output voltage of the laser, the output energy and the optimal detonation energy can be obtained (84.1 mJ). The model of the high-speed camera used in this experiment was APHJ-100, the frequency of high-speed camera was 30,000 fps and the laser is a Nd:YAG Dawa-200 single pulse laser with a wavelength of 1064 nm and pulse width of 6 ns.

**Figure 2:**
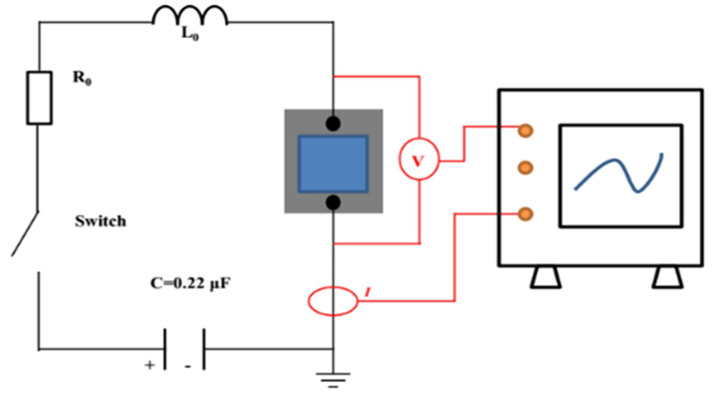
Schematic diagram of the electric explosion experiment device.

An electrical explosion performance test was performed on the energetic film. The schematic diagram of the electrical explosion experiment device is shown in Figure 2. A high-voltage ceramic capacitor was used as the energy storage capacitor. The capacitance was 0.22 μF and the initial explosion voltage was 1500 V. The high-voltage probe and the Rogowski current loop were used to test the voltage and current changes in the energetic film during the ignition process and recorded with an oscilloscope. The electric explosion curve was analyzed.

The circuit was connected first, then the sample was soldered to the positive and negative terminals of the high-voltage output terminal of the printed circuit board (PCB), and the resistance was measured to be 0.4 Ω. The high voltage (1500 V) of the pulse power source acted on both ends of the high-pressure gas switch, and the trigger signal (3000 V) was output by the pulse power source, thereby turning on the gas switch, completing the high-voltage output and acting on both ends of the sample.

The electrostatic sensitivity test of the silicon-based Cu(N_3_)_2_@ CNTs composite energetic film was carried out by the Bruceton method. We tested the sample at a static ignition energy and ignition voltage to evaluate the electrostatic sensitivity of the sample prepared. The 50% ignition energy (E_50%_) of the sample was calculated by the critical ignition voltage (U_50%_).

## 3. Results

### 3.1. The Silicon-Based Al/Ti Film

**Figure 3:**
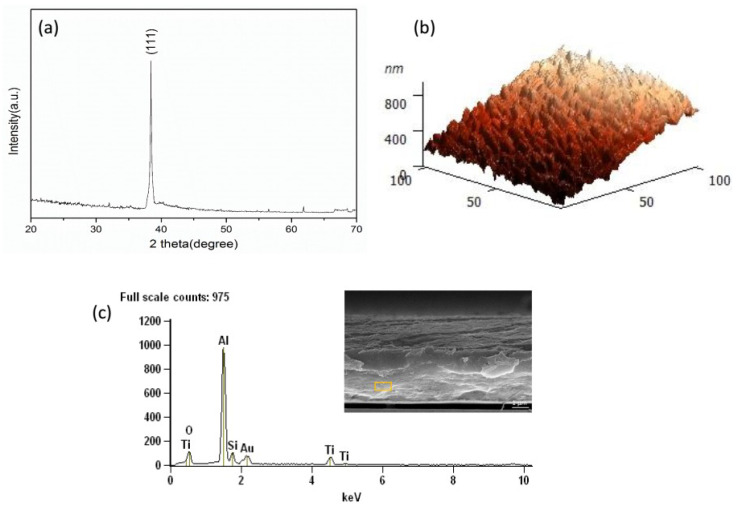
X-ray diffraction (XRD), atomic force microscope (AFM) and energy dispersive X-ray spectroscopy (EDS) patterns of the silicon-based Al/Ti film: (**a**) XRD; (**b**) AFM; (**c**) EDS.

Figure 3a shows the XRD pattern of the silicon-based Al/Ti film. Compared with the standard pattern (PDF#01-1176), it is obvious that when 2θ is 38.53°, the corresponding characteristic peak is the Al (111) plane. The aluminum film grows preferentially on the (111) crystal plane, which indicates that it has the lowest surface free energy in the direction of the (111) crystal plane. As in the atomic force microscope (AFM) diagram shown in Figure 3b, the aluminum film grows in an island-like structure, and the average surface roughness is about 30 nm. Figure 3c shows the EDS spectrum of the sample. The Si peaks, Al peaks and Ti peaks present in the spectrum are from the silicon substrate and the Al layer as well as the Ti layer. The O peak is from the oxidized alumina of the Al layer. The Au peak comes from the gold-spraying operation performed during the sample preparation to increase conductivity.

### 3.2. The Silicon-Based Porous Alumina Film

**Figure 4:**
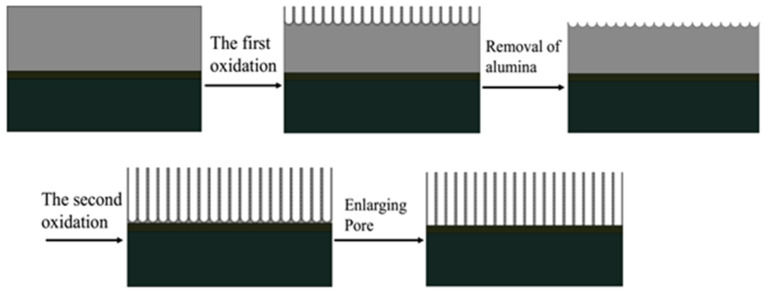
Schematic diagram of the anodizing process.

Figure 4 shows a schematic diagram of the anodizing process. The surface of the Al film prepared by electron beam evaporation deposition is an island-like structure. The first step of anodizing caused some uniformly distributed dents on the surface of the aluminum film. During this process, an alumina film will also be produced. After the alumina film is removed, these dents become the initial sites for the growth of the pores during the second step of anodizing, allowing highly ordered pores to grow. After undergoing the pore enlarging process, the diameter of these channels becomes larger, so it is more suitable as a template for depositing carbon nanotubes.

**Figure 5:**
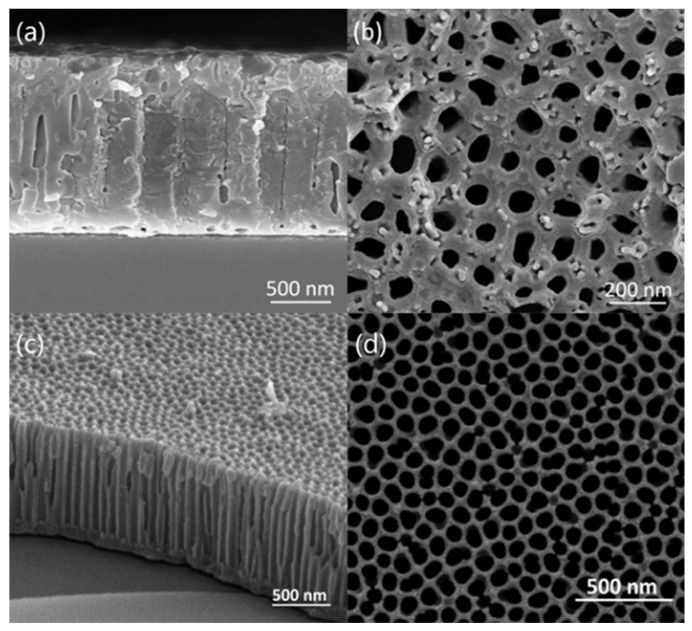
Field emission scanning electron microscopy (FESEM) images of the silicon-based porous alumina film: (**a**) section, before pore enlarging; (**b**) surface, before pore enlarging; (**c**) section, after pore enlarging; (**d**) surface, after pore enlarging.

As depicted in Figure 5, the cross-section and surface FESEM images of the porous alumina film indicate the formation of a porous structure. Figure 5a,b shows the FESEM of the film before pore enlarging. As shown in Figure 5a, the thickness of the well-organized porous alumina membranes (PAM) is about 1.2 μm. Due to the increase in the oxidation time in the first step, the surface of the PAM is relatively flat (Figure 5b). Both the pore diameter and the pore spacing are 100 nm approximately. Figure 5c,d shows a cross-section and surface FESEM image of the PAM in a 5 wt% phosphoric acid solution at 30 °C for 40 min. After the pore treatment, the barrier layer of the alumina film was basically removed, indicating that the barrier layer was effectively removed during the pore enlarging process. The pore diameter of the PAM becomes larger and the diameter is 150 nm approximately and relatively regular and uniform (Figure 5d).

### 3.3. The Silicon-Based Cu@CNTs Composite Film

Carbon nanotube arrays can be prepared by chemical vapor deposition through the template method. In this process, acetylene served as a source of carbon. The carbon produced by the cracking of acetylene was deposited on the inner surface of the channel. Acetylene was continuously introduced into the tube furnace to ensure the integrity of the deposition process. Moreover, the alumina template as a catalyst improved the efficiency of the carbon nanotube growth process.

**Figure 6:**
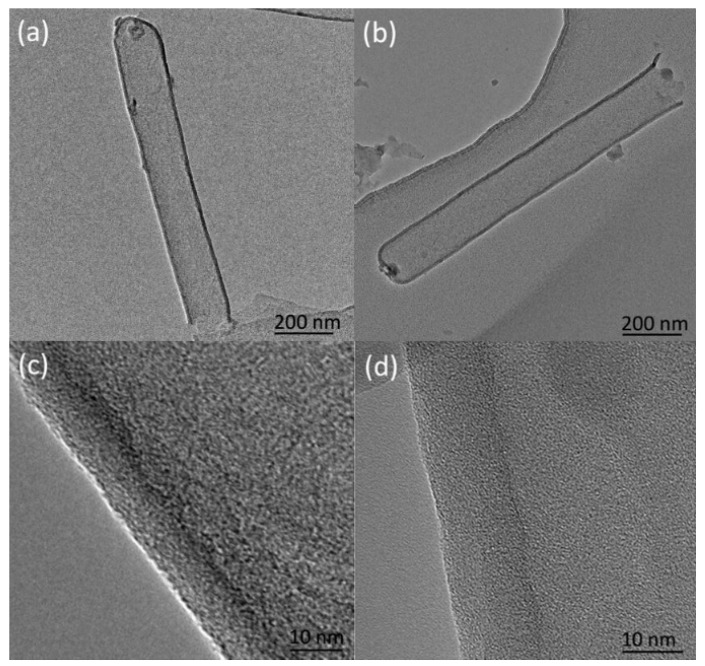
Transmission electron microscopy (TEM) and high-resolution transmission electron microscopy (HRTEM) images of carbon nanotubes prepared at different deposition times: (**a**) TEM, 1.5 h; (**b**) TEM, 2 h; (**c**) HRTEM, 1.5 h; (**d**) HRTEM, 2 h.

Figure 6 shows TEM images of the carbon nanotubes prepared at different deposition times. Figure 6a,c shows the TEM of the carbon nanotubes with a deposition time of 1.5 h, and Figure 6b,d shows the carbon nanotubes with a deposition time of 2 h. Carbon nanotubes replicate the pores of the PAM well and their outer diameter is basically the same as that of the alumina film, about 150 nm (Figure 6a,b). Its length is basically the same as the thickness of the alumina film, which is about 1.2 μm. The wall thickness of the carbon nanotubes in Figure 6c is 9–10 nm, and in Figure 6d is 13–14 nm. As the reaction time increases, the wall thickness increases accordingly. Since the crystallinity of the wall of the carbon nanotubes is poor, it is difficult to identify the number of layers of the carbon nanotubes.

**Figure 7:**
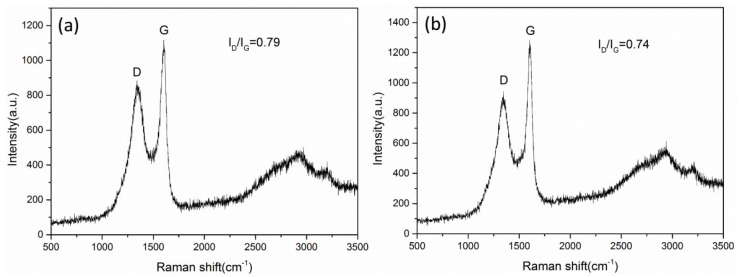
Raman spectra of carbon nanotubes when the deposition time is 1.5 h (**a**) and 2 h (**b**).

Figure 7a,b shows the Raman spectra of the carbon nanotubes when the deposition time is 1.5 h and 2 h, respectively. The I_D_/I_G_ were 0.79 and 0.74, indicating that the crystallinity of the carbon nanotubes was poor. It shows that the crystallinity of the carbon nanotubes does not change significantly with the increase in deposition time.

The addition of organic additives has a great impact on the size, morphology and crystal orientation of the electrodeposited particles and they can be used alone or in combination. In this work, 0.08 mol/L copper sulfate pentahydrate and 0.4 mol/L boric acid were used as the basic electrolytes. The following cases were compared: (a) no additives, (b) thiourea (TU) with a concentration of 0.01 g/L and (c) 0.01 g/L of TU and 0.1 g/L of polyvinylpyrrolidone (PVP). The electrochemical deposition time was 2 h. After the experiment, the products were characterized by TEM.

**Figure 8:**
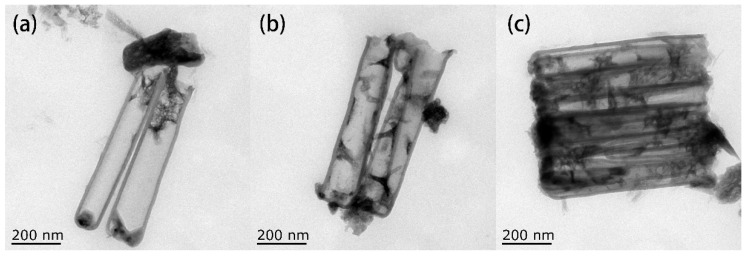
TEM images of copper-filled carbon nanotubes (CNTs) with different additives: (**a**) None; (**b**) thiourea (TU); (**c**) TU and polyvinylpyrrolidone (PVP).

Figure 8 shows the TEM images of these products. Figure 8a is the morphology of the sample without the additives. Obviously, the copper nanoparticles have a larger particle size and are distributed at the mouth of the carbon nanotubes, blocking the mouth. Figure 8b is the morphology of copper after adding TU at a concentration of 0.01 g/L. It can be found that the density of the copper particles in the CNTs is low. Figure 8c shows the morphology of copper deposited in the CNTs after adding both TU and PVP. Compared with Figure 8b, the deposition density of the copper nanoparticles in the CNTs has increased.

**Figure 9:**
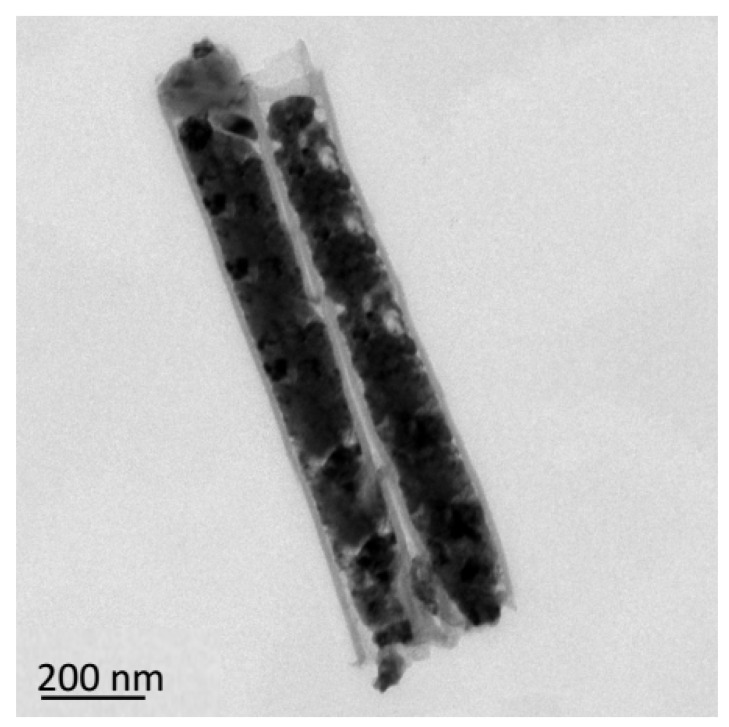
TEM image of the sample after 24 h in air.

The sample was placed in the air for 24 h, and its morphology is shown in Figure 9. Due to the rapid oxidation rate of the deposited copper nanoparticles in the air, which turns the copper into cuprous oxide, the morphology of the copper nanoparticles deposited in the CNTs has changed. In order to demonstrate the composition of the products, the products before and after the change were characterized.

**Figure 10:**
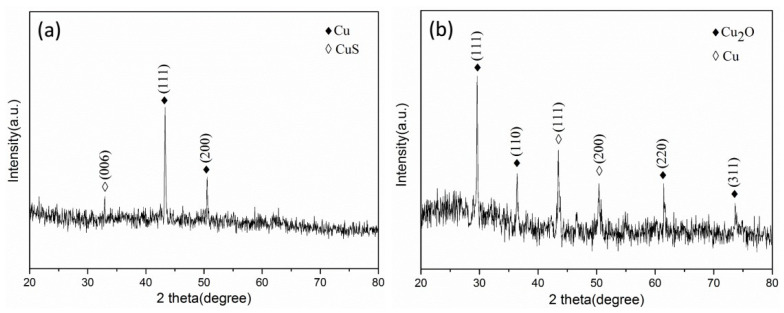
XRD patterns of samples under different storage conditions: (**a**) stored in absolute ethanol; (**b**) exposed to air for 24 h.

Figure 10a,b shows the XRD patterns of the samples stored in absolute ethanol and left in the air for 24 h, respectively. Comparing Figure 10a with the standard spectrum (PDF#01-1241), there are obvious characteristic diffraction peaks of copper between 20° and 80°. When 2θ is located at 43.317° and 50.52°, it corresponds to the (111) and (200) crystal planes of copper. The peak of the (111) crystal plane of copper is stronger than that of the (200) crystal plane, which indicates that copper preferentially grows on the (111) crystal plane. Compared with the standard spectrum (PDF#65-3561), it can be confirmed that the diffraction peak at 32.931° is the (006) crystal plane of CuS. Comparing Figure 10b with the standard spectrum (PDF#65-3288), it can be seen that there are obvious characteristic diffraction peaks of cuprous oxide. The diffraction peaks at 29.562°, 36.419°, 61.454° and 73.678° correspond to the (111), (110), (220) and (311) crystal planes of cuprous oxide, respectively. Compared with the standard spectrum (PDF#01-1241), the diffraction peaks at 43.528° and 50.425° correspond to the (111) and (200) crystal planes of copper, respectively, and the diffraction peaks of copper become weaker. It shows that copper is oxidized to cuprous oxide.

**Figure 11:**
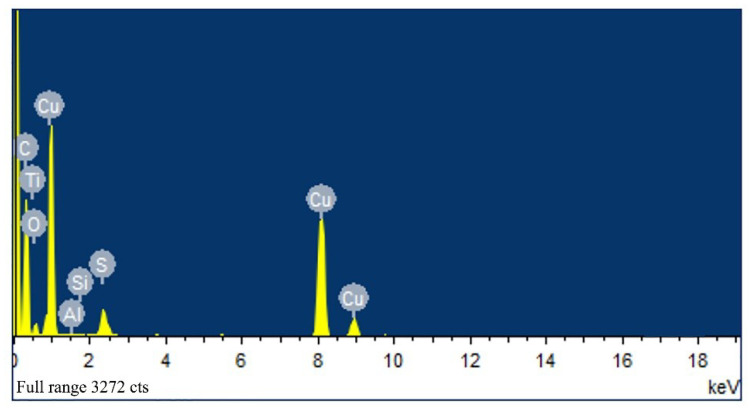
EDS spectrum of the sample stored in absolute ethanol.

Figure 11 is an EDS spectrum of a sample stored in absolute ethanol. There are Si, Al, O, Cu, C, Ti and S peaks in the figure. The Si and Ti peaks come from the silicon substrate and titanium layers. The Al and O peaks are very weak, they come from the PAM which has not been completely removed. The C peak comes from carbon nanotubes, while the Cu peak is nano-copper particles. The existence of the S peak indicates that the TU and copper ions have formed sulfides. Compared with the XRD pattern, it can be concluded that the S element is derived from CuS, and it is confirmed that the sulfur ions in thiourea and copper ions in the electrolyte form copper sulfide.

### 3.4. The Silicon-Based Cu(N_3_)_2_@CNTs Composite Energetic Film

**Figure 12:**
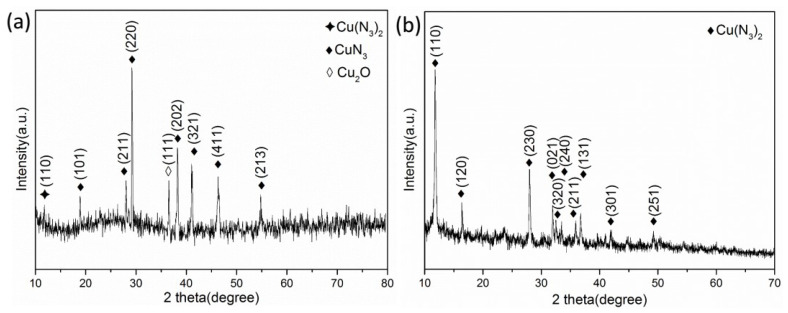
XRD spectra of the azide products of (**a**) the Cu_2_O@CNTs film and (**b**) the Cu@CNTs film.

Figure 12a,b shows the XRD spectra of the azide products of the Cu_2_O@CNTs composite film and Cu@CNTs composite film, respectively. The characteristic diffraction peaks of cuprous azide appear in Figure 12a. Compared with the standard spectrum (PDF#04-0622), the diffraction peaks at 18.927°, 28.002°, 29.162°, 38.237°, 41.060°, 46.304° and 54.774° correspond to the (101), (211), (220), (202), (321), (411) and (213) crystal planes of cuprous azide, respectively. Compared with the standard spectra (PDF#65-3288) and (PDF#21-0281), it can be found that the diffraction peaks at 36.523° and 11.767° correspond to the (111) crystal plane of cuprous oxide and (110) crystal plane of copper azide. The copper azide diffraction peak is very weak, which shows that cuprous oxide mainly formed cuprous azide after the azide reaction. The diffraction peak of copper azide is obvious in Figure 12b. Compared with the standard spectrum (PDF#21-0281), the diffraction peaks at 11.805°, 16.366°, 27.942°, 31.916°, 32.462° and 33.436°correspond to the (110), (120), (230), (021), (320), (240), (211), (131), (301) and (251) crystal planes of copper azide, respectively. It shows that copper was mainly converted into copper azide after the azide reaction, and no cuprous azide was formed.

**Figure 13:**
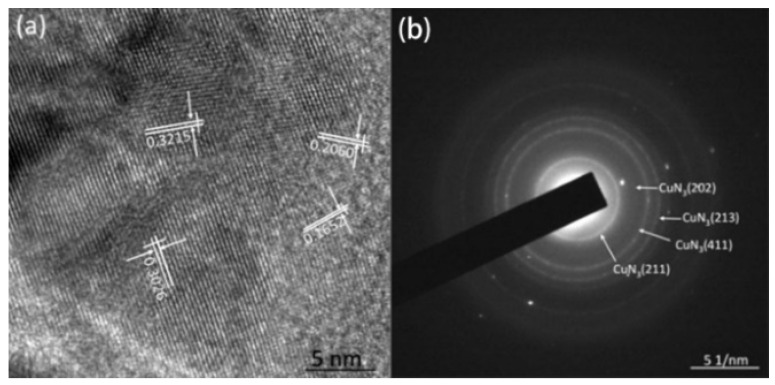

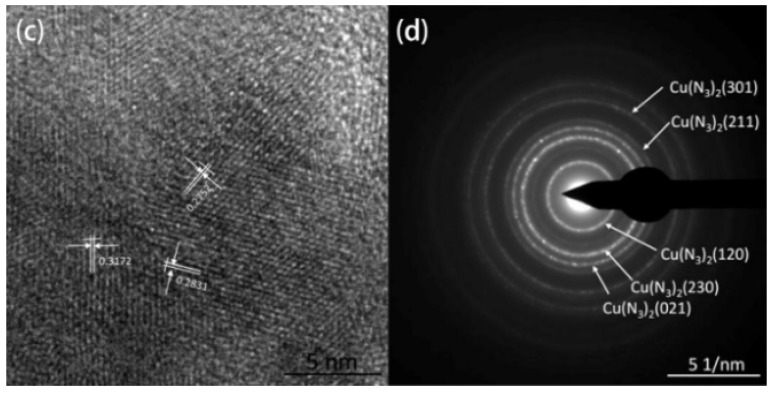
(**a**) HRTEM and (**b**) selective electron diffraction (SAED) images of the azide product of the Cu_2_O@CNTs film; (**c**) HRTEM and (**d**) SAED images of the azide product of the Cu@CNTs film.

Furthermore, the azide products were analyzed by HRTEM and SAED. Figure 13a,b show the HRTEM and SAED images of the azide product of the Cu_2_O@CNTs film. There are clear lattice fringes in Figure 13a. Calculated by the software, the interplanar spacings of the material are 0.3215, 0.3026, 0.2060 and 0.1652 nm, which correspond to the (211), (220), (411) and (213) crystal planes of cuprous azide, respectively (the theoretical values of the interplanar spacing are 0.3180, 0.3060, 0.1960 and 0.1675 nm, respectively). It can be determined from SAED that the product is mainly cuprous azide. Figure 13c,d shows the HRTEM and SAED images of the azide product of the Cu@CNTs film. The crystal plane spacings in the figure are 0.3172, 0.2831 and 0.2752 nm, respectively, corresponding to the (230), (021) and (320) crystal planes of copper azide (the theoretical values of the crystal plane spacing are 0.3190, 0.2800 and 0.2762 nm), and the electron diffraction ring in SAED also confirmed that the product is mainly copper azide.

### 3.5. Properties of Composite Energetic Films

**Figure 14:**
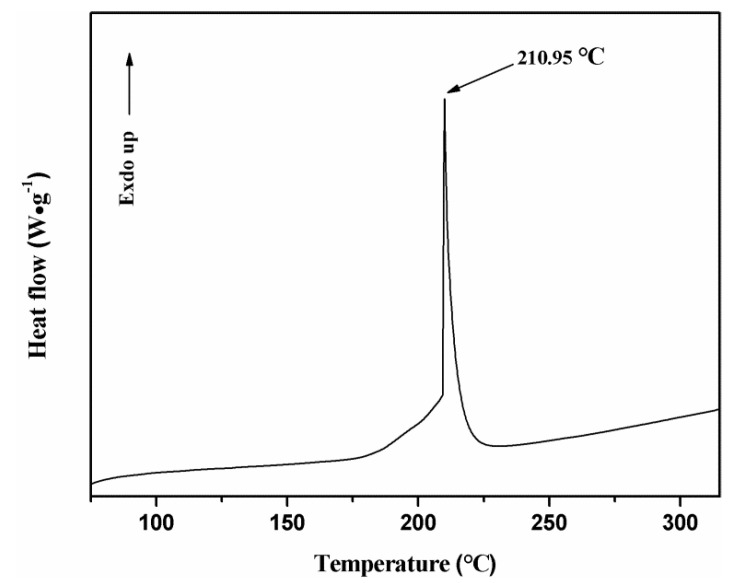
Differential scanning calorimetry (DSC) result of the copper azide filled CNTs energetic film.

DSC was used to analyze the thermal behavior of the Cu(N_3_)_2_@CNTs composite film. As depicted in Figure 14, the exothermic temperature of the composite energetic material is 168.79 to 223.75 °C and a sharp exothermic peak was observed at 210.95 °C, indicating a rapid explosion reaction of the energetic film.

The electrostatic sensitivity of the Cu(N_3_)_2_@CNTs energetic film was tested by the Bruceton method. The results are shown in Table 1. The U_50%_ is 4.0 kV and E_50%_ is 4.0 mJ. 

**Figure 15:**
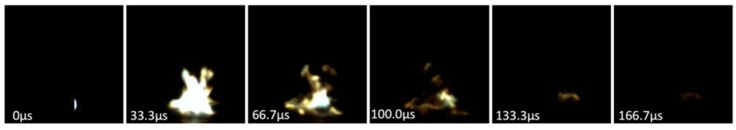
High-speed photography of energetic films during laser ignition.

The energetic film was ignited by laser and the ignition process was recorded by high-speed photography (Figure 15). The area of the flame reached its maximum at 33.3 μs, and the flame lasted about 166.7 μs.

**Figure 16:**
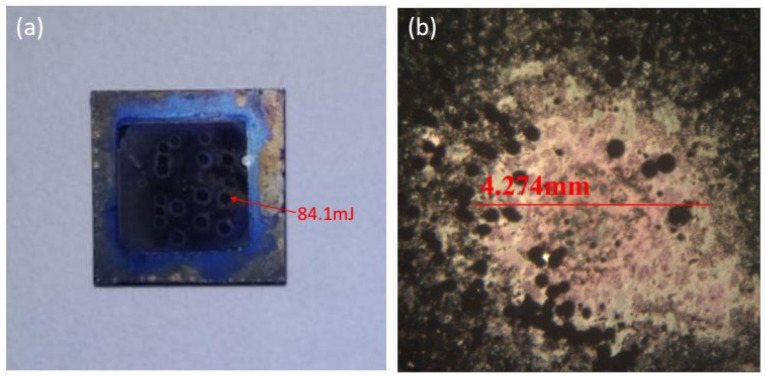
The appearance of the film after laser ignition: (**a**) physical photo; (**b**) optical microscope photo (32 times).

Figure 16 shows the appearance of the film after laser ignition. The diameter of the firing area is about 4.274 mm. The firing position is concave. Spattering can be observed around it and black spots appear, which indicates that the film was melted during laser ignition.

**Figure 17:**
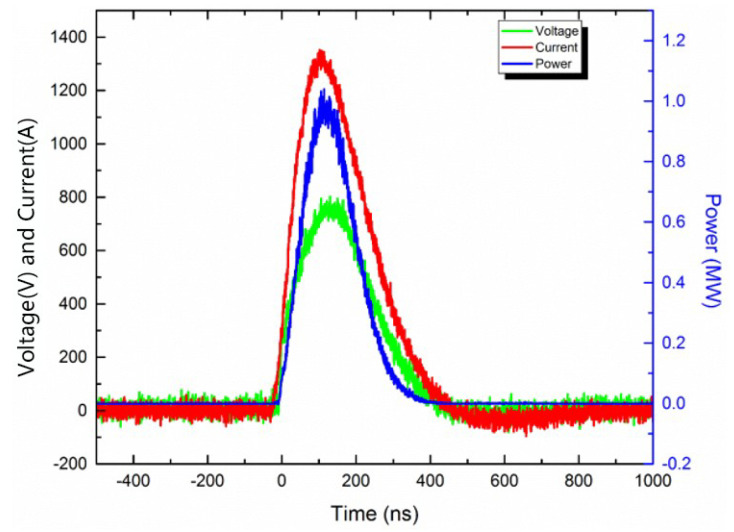
The electric explosion characteristic curve of the energetic film.

Figure 17 shows the electric explosion characteristic curve of the energetic film. The energy utilization ratio η is obtained through Equation (3). The parameters such as current and voltage in the curve are listed in Table 2. One of the envisaged applications of the composite energetic film designed in this article is as an initial energy-converting component in micro-detonation devices. The high-energy pulse capacitor gave a certain amount of energy to the composite energetic film, and the composite energetic film exploded to generate energy. The electric explosion characteristic mainly refers to the energy conversion efficiency of the energetic film in this process. The energy utilization ratio η is the ratio of $\int_{0}^{t}U\cdot Idt$ and $1/2CU_{0}^{2}$. $\int_{0}^{t}U\cdot Idt$ is the integral of U·I, and the integral interval is from 0 to *t* (the time when the current reaches the peak value). It is the energy obtained by the energetic film from the capacitor during the explosion. $1/2CU_{0}^{2}$ is the energy stored in the high-voltage capacitor. The value of η shows that the energetic film has a good energy conversion ability during the electrical explosion process. Moreover, the time for the current to rise to the peak value is short, and the peak value of the current is large. These show that the energetic film has good electrical explosion properties. Therefore, it is expected to be used as a kind of energy conversion component in the micro-detonation device.

(3) $$\eta =\frac{\int_{0}^{t}U\cdot Idt}{1/2CU_{0}^{2}}$$

## 4. Discussion

In this work, carbon nanotube arrays were prepared by chemical vapor deposition and template methods. Levin et al. [29] analyzed the existence form of an anodic aluminum oxide (AAO) template at different temperature stages during heating. When the temperature is above 1200 °C, the existence form of AAO is stable α-Al_2_O_3_. When the temperature reaches 230–500 °C, it changes to η-Al_2_O_3_, and the existence form is mainly γ-Al_2_O_3_ when the temperature is 450–750 °C. The η-Al_2_O_3_ and γ-Al_2_O_3_ have higher porosity and a large specific surface area, so they have better catalytic activity. The commonly used carbon source gas can only be thermally decomposed at high temperatures (800 °C). When acetylene was used as the carbon source, the aligned carbon nanotube array can be prepared in the AAO template channel at about 600 °C. It shows that the AAO template plays a catalytic role in the thermal decomposition of acetylene, so that it can be decomposed at lower temperatures. Liu et al. [30] studied that the catalytic process of the AAO template has two main steps. The first is the catalytic effect when the carbon source is decomposed, and the second is the catalytic effect during the growth of carbon nanotubes.

**Figure 18:**
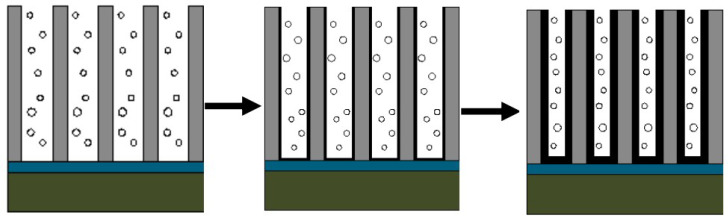
Schematic diagram of the mechanism for preparing carbon nanotube arrays through porous alumina membranes (PAM).

Yang et al. [31] studied the growth mechanism of carbon nanotubes prepared by the AAO templates and found that carbon atoms were deposited on the walls of the alumina template and the channels of the AAO template were copied to obtain the carbon nanotube structure. The carbon nanotubes are named a-CNTs (amorphous carbon nanotubes). By analyzing the Raman spectrum and TEM, it can be known that the carbon nanotubes obtained in this work are also a-CNTs. With reference to Yang’s conclusion, we consider the mechanism of preparing carbon nanotube arrays through PAM, as shown in Figure 18.

First, the carbon source is cracked to generate carbon atoms under the catalysis of the PAM. Carbon atoms are deposited in the pores of the PAM. Under the autocatalysis of the PAM, the carbon atoms replicate the pore structure of the PAM and grow into carbon nanotubes at a relatively lower temperature. The carbon atoms generated by the continuous decomposition of the carbon source continue to enter the hollow lumen of the carbon nanotubes, deposit on the inner wall of the formed carbon nanotubes and repeat the growth of the carbon nanotubes. With the increase in the reaction time, the thickness of the carbon nanotube wall also increases and even solid carbon nanorods may be obtained. Therefore, carbon nanotubes of different thicknesses can be obtained by controlling the reaction time.

During the stage of electrochemical deposition, the type of additive has a great influence on the filling effect of the copper nanoparticles. Additives can be generally classified into three categories: halide ions, supporting electrolytes and organic additives. Xu [32] found that the addition of Cl^−^ in the electrolyte would form a “chlorine bridge” under enhanced polarization conditions, changing the charge transfer mechanism of the charged particles in the solution. The role of the supporting electrolyte is generally considered to increase the charge transfer rate and the conductivity of the electrolyte.

By comparing the different morphologies of copper deposited in CNTs, it is obvious that when no additives were added, the crystal size of copper grew too large, resulting in the inability of copper nanoparticles to be deposited in CNTs. When TU was added, the particle size of the copper nanoparticles decreased significantly. These results confirm that TU as an additive has the effect of inhibiting the growth of copper particles. First, TU adsorbs on the surface of the copper particles and reacts with Cu^2+^ to form compounds. These compounds become adsorption layers and cover the active sites of copper, preventing further reduction of Cu^2+^. Then TU and CuS generated by Cu^2+^ are also adsorbed on the surface of the copper particles, making the copper atoms unable to reach the active site and inhibiting the growth of grains. When TU and PVP were added together, the particle size of the copper nanoparticles became smaller, indicating that the inhibitory effect is more pronounced when PVP and TU work together.

**Figure 19:**
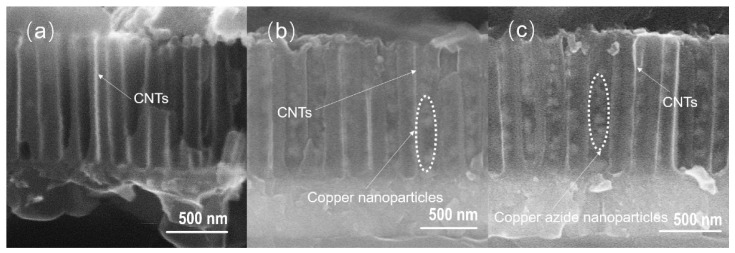
SEM images of the cross-section of (**a**) CNTs grown on silicon-based porous alumina films; (**b**) the silicon-based Cu@CNTs film and (**c**) the silicon-based Cu(N_3_)_2_@CNTs film.

Figure 19 shows the SEM images of the cross-section of CNTs grown on silicon-based porous alumina films, silicon-based Cu@CNTs film and silicon-based Cu(N_3_)_2_@CNTs film. It can be seen from Figure 19a that the carbon nanotubes were tightly attached to the inner walls of the pores of the porous alumina template, and the shape of the pores was well reproduced. Its upper end was open, providing an entrance for the deposition of the copper nanoparticles. Figure 19b shows the cross-section of the film after the copper nanoparticles were filled. The copper nanoparticles were relatively evenly distributed in the carbon nanotubes, without obvious agglomeration. After the azide reaction, the copper nanoparticles were converted to energetic copper azide (Figure 19c). It can be seen that its particles’ size become slightly larger, and the ordered directional carbon nanotube arrays have not been destroyed during the azide process.

Carbon nanotubes have excellent electrical conductivity. When the copper azide is filled into the hollow cavity of the oriented carbon nanotube, the electrostatic charge will be dispersed on the surface of the carbon nanotube instead of accumulating on the surface of the copper azide. Therefore, the explosion of copper azide caused by the electrostatic charge can be avoided, and the electrostatic sensitivity of the composite energetic film can be effectively reduced. Compared with the E_50%_ of pure nano-copper azide (0.05 mJ) reported before [33], the electrostatic sensitivity of the composite film is significantly reduced.

In future research, it will be an interesting direction to combine carbon materials with different structures and copper azide to obtain energetic materials with good performance. Copper azide can take advantage of its high energy density in composite materials, and carbon materials with different structures can isolate copper azide particles in various ways, thereby reducing the mechanical sensitivity of composite energetic materials. Meanwhile, the excellent conductivity of carbon materials can also reduce the electrostatic sensitivity of composite energetic materials. On the one hand, the advantages of copper azide are used, and on the other hand, carbon materials are used to make up for the shortcomings of copper azide as a primary explosive. The composite energetic films designed and prepared in this paper will also be further optimized to be applied to MEMS pyrotechnic devices. Furthermore, an in-depth study of the mechanism of azide reaction will also be a valuable work.

## 5. Conclusions

Energetic films realized by encapsulating copper azide in silicon-based carbon nanotube arrays were presented in this study, which opens a route to integrate primary explosives realized on Ti-coated silicon wafers. A silicon-based alumina template was prepared using a modified anodization process. We prepared CNTs by chemical vapor deposition using the prepared template. Copper nanoparticles were embedded into the carbon nanotubes by means of electrochemical deposition. The prepared Cu@CNTs composite film reacted with hydrogen azide gas to synthesize the copper azide@CNTs composite energetic film with low electrostatic sensitivity. Moreover, this composite film stores a great quantity of energy and can be released rapidly. Therefore, it has great potential to replace the commonly used lead-containing detonator and achieve the miniaturization of the ignition device.

## Figures and Tables

**Table 1:** Electrostatic sensitivity of the silicon-based Cu(N_3_)_2_@carbon nanotubes (CNTs) composite film.

Electrode Gap/mm	Capacity/pF	*U*_50%_/kV	*E*_50%_/mJ
0.12	500	4.0	4.0

**Table 2:** Performance parameters of the electric explosion experiment.

Peak Current/kA	Peak Voltage/kV	Peak Current Time/ns	Peak Voltage Time/ns	Explosion Time/ns	Explosion Power/MW	Explosion Energy/mJ	η/%
1.35	0.80	102.0	130	114.0	1.04	68.61	27.72
